# Supplementary material for: Clinical and Virological Features of SARS-CoV-2 Variants during the Four Waves of the Pandemic in the Mexican Southeast
Source: Trop Med Infect Dis. 2023 Feb 23;8(3):134. doi: 10.3390/tropicalmed8030134 (PMC10053031; doi:10.3390/tropicalmed8030134)
Supplement: Supplementary file 1 [file tropicalmed-08-00134-s001.zip › tropicalmed-2159083-supplementary.pdf]

Supplementary Materials

Table S1. COVID-19 symptoms among vaccination status.

		Vaccination status			
		**No vaccination**	**Incomplete**	**Complete**	***p***
Symptoms (mean ± EE)	6.4 ± 3.0	6.6 ± 3.1	5.6 ± 2.3	5.5 ± 2.3	0.216
Fever (%, n)	72.3% (n = 128)z	73.4% (n = 105)	40.0% (n = 2)	72.4% (n = 21)	0.260
Cough (%, n)	85.9% (n = 152)	86.0% (n = 123)	80.0% (n = 4)	86.2% (n = 25)	0.929
Odynophagia (%, n)	28.2% (n = 50)	32.9% (n = 47)	0% (n = 0)	10.3% (n = 3)	**0.018***
Dyspnea (%, n)	75.7% (n = 134)	76.2% (n = 109)	60.0% (n = 3)	75.9% (n = 22)	0.723
Diarrhea (%, n)	16.4% (n = 29)	17.5% (n = 25)	60.0% (n = 3)	3.4% (n = 1)	**0.005***
Chill (%, n)	7.9% (n = 14)	9.8% (n = 14)	0% (n = 0)	0% (n = 0)	0.164
Headache (%, n)	76.3% (n = 135)	76.9% (n = 110)	80.0% (n = 4)	72.4% (n = 21)	0.856
Myalgia (%, n)	46.9% (n = 83)	46.2% (n = 66)	20% (n = 1)	55.2% (n = 16)	0.320
Joint pain (%, n)	44.6% (n = 79)	44.8% (n = 64)	0% (n = 0)	51.7% (n = 15)	0.099
Runny nose (%, n)	22.6% (n = 40)	23.8% (n = 34)	0% (n = 0)	20.7% (n = 6)	0.442
Anosmia (%, n)	10.2% (n = 18)	10.5% (n = 15)	20.0% (n = 1)	6.9% (n = 2)	0.642
Ageusia	10.2% (n = 18)	11.2% (n = 16)	20.0% (n = 1)	3.4% (n = 1)	0.345

* denotes a significant difference

Table S2. Comparison of cycle threshold value and defunction by pandemic waves.

Gene		**Defunction**	**Wave 1**	**Wave 2**	**Wave 3**	**Wave 4**	***p***
RdRp gene	Low CT	Yes	66.7% (n = 6)	50% (n = 14)	66.7% (n = 7)	43.8% (n = 14)	0.576
		No	33.3% (n = 3)	50% (n = 14)	33.3% (n = 7)	25.9% (n = 7)	
	High CT	Yes	66.7% (n = 18)	63.4% (n = 26)	47.6% (n = 10)	42.9% (n = 15)	0.158
		No	33.3% (n = 9)	36.6% (n = 15)	52.4% (n = 11)	57.1% (n = 20)	
N gene	Low CT	Yes	58.3% (n = 14)	50.0% (n = 12)	55.0% (n = 11)	63.6% (n = 14)	0.821
		No	41.7% (n = 10)	50.0% (n = 12)	45.0% (n = 9)	36.4% (n = 8)	
	High CT	Yes	83.3% (n = 10)	50.0% (n = 6)	38.5% (n = 5)	43.8% (n = 14)	0.089
		No	16.7% (n = 2)	50.0% (n = 6)	61.5% (n = 8)	56.2% (n = 18)	
E gene	Low CT	Yes	63.6% (n = 21)	55.6% (n = 15)	66.7% (n = 10)	63.3% (n = 19)	0.879
		No	36.4% (n = 12)	44.4% (n = 12)	33.3% (n = 5)	36.7% (n = 11)	
	High CT	Yes	100% (n = 3)	37.5% (n = 3)	38.5% (n = 5)	40% (n = 10)	0.233
		No	0% (n = 0)	62.5% (n = 5)	61.5% (n = 8)	60% (n = 15)	
